# Measuring complexity in Brazilian economic crises

**DOI:** 10.1371/journal.pone.0173280

**Published:** 2017-03-16

**Authors:** Letícia P. D. Mortoza, José R. C. Piqueira

**Affiliations:** Laboratório de Automaçãoe Controle/Escola Politécnica, Universidade de São Paulo, Avenida Prof. Luciano Gualberto, travessa 3 - 158, São Paulo-Brazil; University of Rijeka, CROATIA

## Abstract

Capital flows are responsible for a strong influence on the foreign exchange rates and stock prices macroeconomic parameters. In volatile economies, capital flows can change due to several types of social, political and economic events, provoking oscillations on these parameters, which are recognized as economic crises. This work aims to investigate how these two macroeconomic variables are related with crisis events by using the traditional complex measures due to Lopez-Mancini-Calbet (LMC) and to Shiner-Davison-Landsberg (SDL), that can be applied to any temporal series. Here, Ibovespa (Bovespa Stock Exchange main Index) and the “dollar-real” parity are the background for calculating the LMC and SDL complexity measures. By analyzing the temporal evolution of these measures, it is shown that they might be related to important events that occurred in the Brazilian economy.

## 1 Introduction

For the last forty years, complex thinking has influenced many research areas [1] contributing to model problems related to the onset of surprising or catastrophic events such as traffic jams, crowd behavior, tsunamis and earthquakes [2].

Consequently, the concept of complexity has been largely debated [3], with generality implying the prevalence of qualitative arguments [4]. In this scene, concerning to computer science, Shannon [5] and Kolmogorov [6] proposed similar and useful complexity measures.

In terms of economic applications, fractal dimension measures were proposed by Mandelbrot [7], which are largely used to characterize economic time series [8], including different fractal measures adaptable to different patterns of series [9].

Another well spread line of reasoning applied to economic problems is the Dynamical System’s approach, aiming to detect order and chaos in processes [10].

Although the common sense is that complexity is a relative concept [11], López-Ruiz, Mancini and Calbet proposed a global measure for complexity by using the entropy definition, which is referred to as the LMC measure [12].

Additionally, Shiner, Davison and Landsberg, by slightly changing the LMC definition, proposed the SDL measure [13] and both, LMC and SDL, are satisfactory to measure complexity for many problems [14].

Concerning economy, these measures are barely used [15] although they could be useful to model crises by the analysis of their temporal evolutions [16], as it was shown in former work; moreover they could be used as an alternative to the analysis of the tails of statistical distributions [8, 17].

Here, slightly modified versions of the LMC and SDL measures are proposed for temporal series. Then, considering that capital fluxes are determinant to the economic health of an emerging country [18], oscillating drastically due to several political, social and economic changes, Brazilian macro-economical data are analyzed by calculating the LMC and SDL complexity measures, aiming to identify how their behavior is connected to crisis events.

The chosen data are Ibovespa (Bovespa Stock Exchange main Index) and the “dollar-real” parity, generating the LMC and SDL complexity measures temporal series that are studied in five critical periods of the Brazilian economy. Since the end of dictatorship regime in 1985, Brazil has been alternating short cycles of economical phases, with macroeconomics indicators oscillating, apparently commanded by the political changes.

To delineate the Brazilian political events, the analysis starts with José Sarney’s term, between 1985 and 1990, which is considered the transition period between dictatorship and democracy and in which several stabilization plans were practiced, such as freezing prices, causing an increase in demand and a strong monetary black market. In 1990, Fernando Collor de Mello was democratically elected and applied the “Collor” plan, radically freezing the financial assets, creating the “commercial-dollar”, a parallel to the existing fluctuating rates. Latter, the “Collor” plan-II was enforced based on freezing prices [17].

The Collor plans promoted important changes in the Brazilian economy, mainly opening the market to foreign capital and privatizing important service companies formerly controlled by the state. Corruption suspicions and a bad economic scenario caused the impeachment of the president (Fernando Collor de Mello) who was replaced in September, 1992. A new stabilization plan was designed (“Real” plan), and started to be applied in 1993, with a new currency, linked to the dollar quotation and implying a strong inflation decrease [17].

The exchange policy was pressed and, in 1999, the Brazilian currency was finally allowed to have free fluctuations, with a strong initial devaluation. The data sampling presented here starts from this period on, considering that the economic policy did not suffer any additional change.

Between 1999 and 2003, liberal ideas, mainly represented by the new president Fernando Henrique Cardoso, governed the country and a satisfactory economic equilibrium was reached; however, with timid social actions. Supported by ideas regarding social justice and the democratic distribution of gains, the Labor Party, represented by Luiz Inacio Lula da Silva was elected in 2003, governing up to 2011.

This period was characterized by optimism and, consequently, the Labor Party, represented by Dilma Roussef, was elected again in 2011 and in 2016. During Dilma’s second term, many corruption scandals erupted provoking her impeachment in October of 2016. The new period presented no political novelty, with important names of the Senate and the House of Representatives supporting the impeachment also being involved in corruption.

Aiming to verify how political events changed the Brazilian economical life, between 1999 and 2015, this article is divided into five sections, including this introduction. The next section describes the main financial, political and social crisis events that influenced the Brazilian economy in the recent past and its macroeconomics effects. Furthermore, the acquisition of the two time series and the calculation methodology are explained, followed by the presentation of the results. A discussion section presents some conjectures regarding the methodology and the Brazilian crisis events followed by a brief conclusion.

## 2 Crisis events

This analysis starts in January 1999, when Brazil finally abandoned the crawling peg exchange rate regime and adopted floating rates. In this period the Brazilian currency, which was already suffering a huge devaluation pressure, depreciated drastically (49.51% from January to March, 1999). In contrast, Ibovespa increased 59.34% in the same period [19]. This foreign exchange crisis is the first event analyzed in this study.

The second crisis event is the Twin Towers attack, in September 2001, when terrorist acts lead to the complete destruction of the World Trade Center in New York and partial destruction of the Pentagon, in Washington. Although it had a short term effect, it caused the American Stock Exchanges closure during one week and raised the investors risk aversion, causing the removal of investment money from the emerging markets.

The third event is specially related to investor confidence. By the middle of 2002, Luiz Inacio Lula da Silva, main leader of Brazilian Labour’s Party, appeared in the polls as the favorite candidate for the Presidental election. The uncertainty about economic policies lead to a dramatic capital outflow, causing the Brazilian currency to reach its biggest devaluation since it started floating in 1999.

The fourth event is the world’s well known sub-prime crisis. It started with sub-prime mortgages defaults—hence its name—in 2007 and in one year, it contaminated global economy. The peak of this crisis happened in September 2008, when the Federal Reserve surprisingly refused to rescue the Lehman Brothers, triggering a historical liquidity crisis in the interbank money market. Consequently, global credit offer disappeared consequently and the whole World entered into a historic economic recession. It was the biggest global financial crisis since 1930.

The fifth event is the reelection presidential campaign of Dilma Roussef, opposing the desires of the Brazilian elite radically against all the social programs, which started during her first term. The data related to the recent impeachment process are not analyzed here, but it may be insightful in the future.

The main objective is to investigate how the LMC and SDL complexity measures change in the two time series representing Brazilian economy: dollar/real ratio and Ibovespa index. The observation of the dynamics of the complexity measures evidences how crisis events give signals to occur.

## 3 Data and methodology

### 3.1 Data

The data collected are constituted of two different time series: the dollar-real exchange rates and the Ibovespa Index, both expressed on a daily basis. The Brazilian Real / American Dollar exchange rate is compiled by the Brazilian Central Bank and it is available on the INTERNET by accessing (http://www4.bcb.gov.br/pec/taxas/port/ptaxnpesq.asp?id=txcotacao), choosing the period and the option “dolar EUA”.

This series is composed of 4,109 data points, from January 1999 to August 2015. The average rate of this period was 2.2017 BRL/USD, with a 0.4811 standard deviation. The minimum value of 1.2071 BRL/USD occurred in January 1999 and the series reached its maximum of 3.9544 BRL/USD during the elections in October 2002.

**Fig 1:**
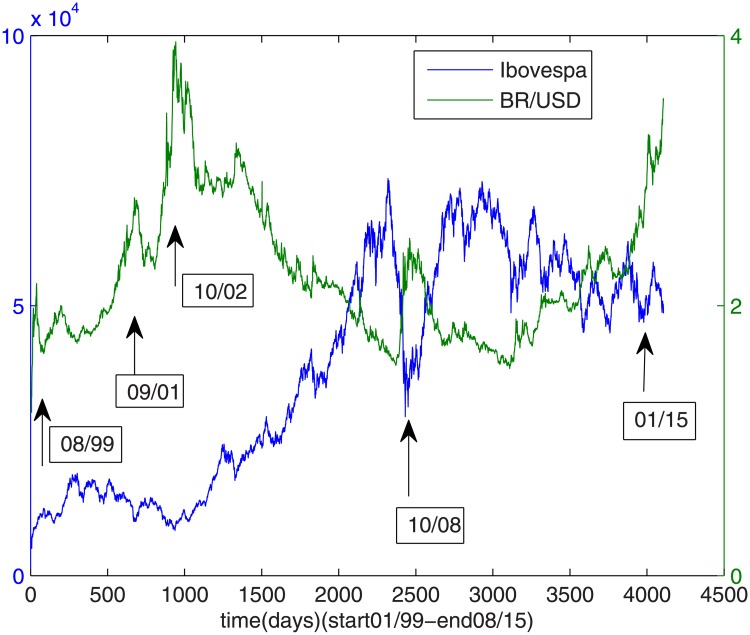
Temporal series: USD/BRL and Ibovespa.

This time series is plotted in Fig 1, where five local maxima can be spotted: March 1999, September 2001, October 2002, October 2008 and January 2015, which are indicated by arrows. These peaks correspond to the 1999 Exchange Rate Crisis, the Twin Tower Crisis, 2002 Election Crisis, Sub-Prime Crisis and the reelection of Dilma Roussef reelection, respectively.

On the other hand, Ibovespa is the main Bovespa (Brazilian Stock Exchange) index. It is compiled as a weighted average of a theoretical portfolio of stocks. It is designed to gauge the average performance of the stock market by tracking changes in the prices of the more actively traded and the better representative stocks of the Brazilian stock market. Ibovespa is a total return index and it is expressed in points. It was first compiled in January 1968, when it was represented by 100 points.

As to the foreign exchange rates, 4,109 daily data points, from January 1999 to August 2015, compose this time-series, available on the INTERNET (http://www.bmfbovespa.com.br/pt-br/produtos/indices/indices-amplos/) where the window “Ibovespa” must be opened and “Serie retroativa Ibovespa” must be selected. The average for this period is of 38,140 points, with a standard deviation of 20,720. The series minimum was of 5,057 points in the beginning of the studied period, January 1999; and the maximum of 73,516 points happened in May 2008, months before the peak of the Sub-Prime Crisis.

The Ibovespa time series is also plotted in Fig 1, where its upward trend can be observed, from the beginning of the period until 2008, when it reached its maxima. Following, a huge depression was caused by the Sub-Prime Crisis and the recovery just happened by the end of 2009 and the beginning of 2010. Apart from this trend, the local minima can be spotted around August 1999, September 2001, October 2002, October 2008 and January 2015, which coincides with the crisis events studied here.

### 3.2 Methodology

To calculate the LMC and SDL complexity measures for a temporal series, it is necessary to define the instantaneous order Δ analogously to the definition of disorder based on Shannon entropy [12, 13], demanding the division of the range of the variables into intervals, to calculate the instantaneous probability associated to the measured value.

First both series were divided into *N* = 16 discrete parts, with a 0.175 BRL/USD amplitude for the foreign exchange rate and 4,279 stock points for the Ibovespa. The relative frequency of occurrence of each interval is considered to be its individual probability (*p*_*i*_) and the number *N* was chosen after several numerical experiments optimizing accuracy and computational time.

By using the calculated probabilities, the instantaneous disorder, corresponding to the thermodynamical equilibrium [12, 13], for the interval *H*_*i*_ is given by:

$$\begin{array}{c} \Delta_{i}=p_{i}\frac{\left(-log_{2}p_{i}\right)}{log_{2}N}, \end{array}$$ (1)

and considering the order term (1 − Δ_*i*_), corresponding to the thermodynamical disequilibrium [13], the instantaneous SDL complexity measure is given by:

$$\begin{array}{c} {\left(SDL\right)}_{i}=\Delta_{i}\left(1\;-\;\Delta_{i}\right). \end{array}$$ (2)

As proposed in [12], the LMC complexity is calculated by replacing the order term by a disequilibrium expression [12], given by;

$$\begin{array}{c} D_{i}={\left(p_{i}\;-\;\frac{1}{N}\right)}^{2}; \end{array}$$ (3)

consequently, the instantaneous LMC is given by:

$$\begin{array}{c} {\left(LMC\right)}_{i}=\Delta_{i}D_{i}. \end{array}$$ (4)

The calculations described were performed considering the data represented in Fig 1 and are summarized in Table 1 for the Dollar-Real ratio and in Table 2 for the Ibovespa index.

**Table 1:** Dollar-real ratio.

Interval	Min	Max	Occurrence	Probability (%)	SDL	LMC
H1	1.2071	1.3788	9	0.22	0.00008	0.00000
H2	1.3788	1.5505	4	0.10	0.00004	0.00000
H3	1.5505	1.7222	440	10.71	0.00264	0.00001
H4	1.7222	1.8939	866	21.08	0.00469	0.00002
H5	1.8939	2.0656	571	13.90	0.00329	0.00001
H6	2.0656	2.2373	525	12.78	0.00306	0.00001
H7	2.2373	2.4090	554	13.49	0.00320	0.00001
H8	2.4090	2.5808	220	5.36	0.00145	0.00001
H9	2.5808	2.7525	173	4.21	0.00118	0.00000
H10	2.7525	2.9242	269	6.55	0.00173	0.00001
H11	2.9242	3.0959	204	4.97	0.00136	0.00001
H12	3.0959	3.2676	123	2.99	0.00088	0.00000
H13	3.2676	3.4393	37	0.90	0.00030	0.00000
H14	3.4393	3.6110	65	1.58	0.00050	0.00000
H15	3.6110	3.7827	30	0.73	0.00025	0.00000
H16	3.7827	3.9544	18	0.44	0.00005	0.00000

**Table 2:** Ibovespa index.

Interval	Min	Max	Occurrence	Probability (%)	SDL	LMC
H1	5,057.19	9,335.92	66	1.61	0.00051	0.00000
H2	9,335.92	13,614.64	603	14.68	0.00344	0.00002
H3	13,614.64	17,893.37	496	12.07	0.00292	0.00001
H4	17,893.37	22,172.10	1814	4.41	0.00123	0.00000
H5	22,172.10	26,450.82	255	6.21	0.00165	0.00000
H6	26,450.82	30,729.55	92	2.24	0.00068	0.00000
H7	30,729.55	35,008.27	77	1.87	0.00058	0.00000
H8	35,008.27	39,287.00	227	5.53	0.00149	0.00000
H9	39,287.00	43,565.73	123	2.99	0.00088	0.00000
H10	43,565.73	47,844.45	141	3.43	0.00099	0.00000
H11	47,844.45	52,123.18	313	7.62	0.00197	0.00000
H12	52,123.18	56,401.91	486	11.83	0.00287	0.00001
H13	56,401.91	60,680.63	344	8.37	0.00214	0.00000
H14	60,680.63	64,959.36	308	7.50	0.00194	0.00000
H15	64,959.36	69,238.08	273	6.65	0.00175	0.00000
H16	69,238.08	73,516.81	1237	2.9	0.00088	0.00000

## 4 Results

### 4.1 The whole picture

**Fig 2:**
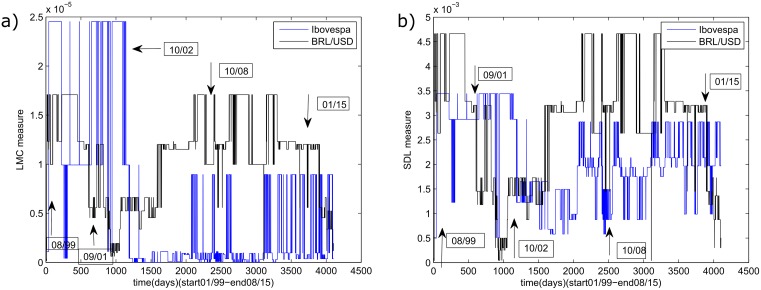
Complexity measures temporal evolutions. (a) LMC complexity measure. (b) SDL complexity measure.

After calculating the LMC and SDL complexity measure for each interval in each series, the data points were placed back into their original sequence to construct the complexity measures time evolution for each variable. Finally both complexity series were combined permitting a visual comparison for each measure (Fig 2a and 2b), with arrows indicating the crises to be studied here.

The figures indicate that the temporal series for the two complexity measures (LMC and SDL) present the same qualitative behavior. Then, for the sake of simplicity, from this point on, only the SDL results are presented.

Another interesting point highlighted is the presence of higher frequency oscillation for the complexity measures coinciding with the five crisis events described in section 2.

### 4.2 Changing currency policies

**Fig 3:**
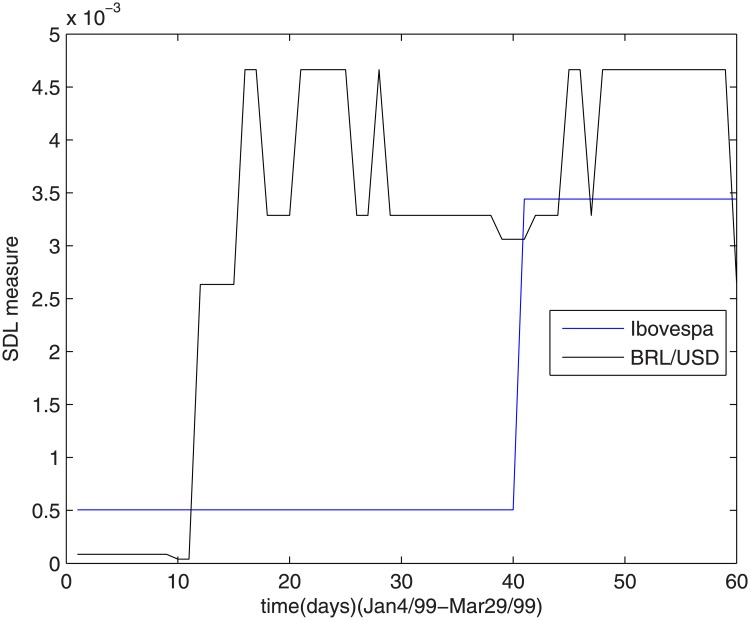
The SDL complexity measure during changes in the Brazilian exchanging regime.

To investigate the described periods of crisis, a zoom is provided for each one, starting with the changes in the Brazilian exchanging regime, shown in Fig 3.

Ibovespa index suffer a strong change from a low to a high value in March-99, a retarded effect from governmental economic actions over currency regulations. The effects over the measures for dollar-real ratio are more sensitive and the complexity measure oscillates during January, February and March of 1999.

### 4.3 Twin towers attack

**Fig 4:**
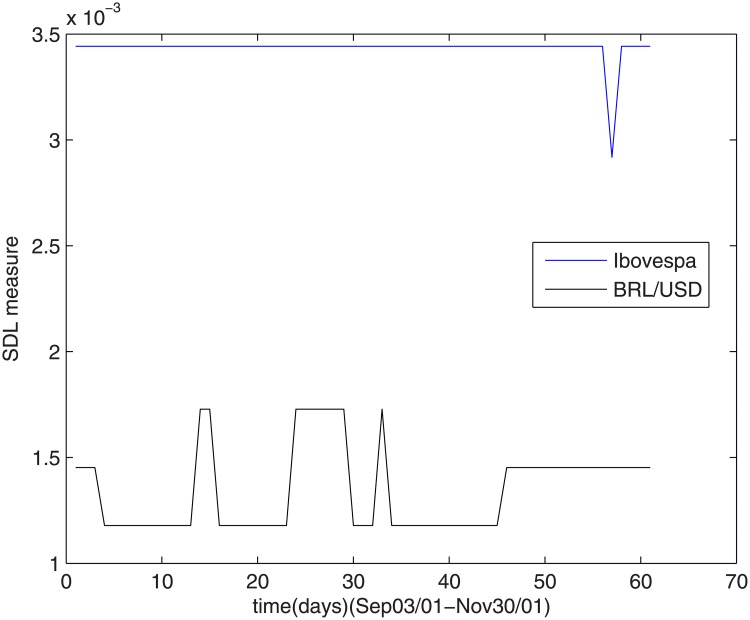
The SDL complexity measure after September 11^th^.

When the September 11^th^ attack occurred, Brazilian stock market suffered very strong effects, as it can be deduced from observing Fig 4, which shows how the complexity measures were affected in this period.

Ibovespa index complex measure remained high and constant in this period, indicating that the closing of American stock agencies produced important uncertainties in the Brazilian stock market. Concerning the dollar-real ratio, the complexity remained constant, but at lower level than of the Ibovespa index.

### 4.4 Lula’s campaign

**Fig 5:**
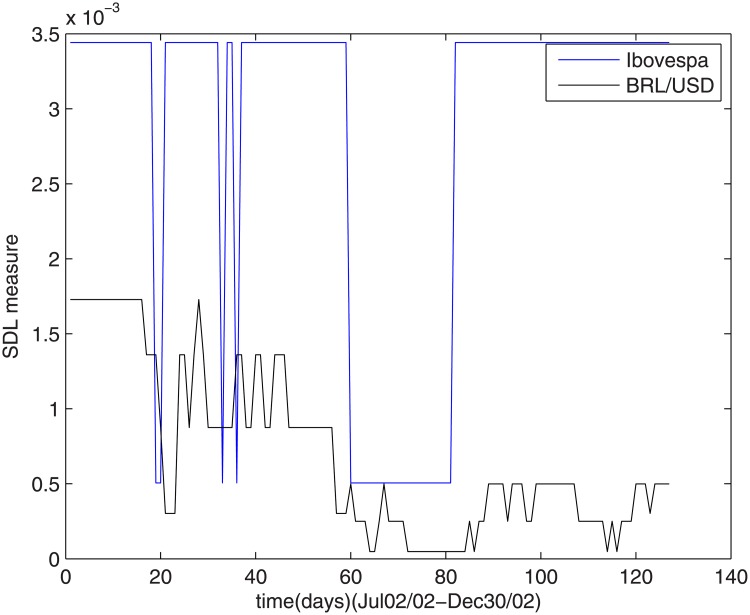
The SDL complexity measure during Lula’s campaign.

The Ibovespa index shows to be more sensitive to Lula’s campaign than the dollar/real ratio, as shown in Fig 5. The Ibovespa index complexity oscillated between high and low levels all over the campaign and remained high after the election results.

The dollar/real ratio oscillated in a lower level, maintaining a practically constant low level complexity.

### 4.5 Sub-prime crisis

**Fig 6:**
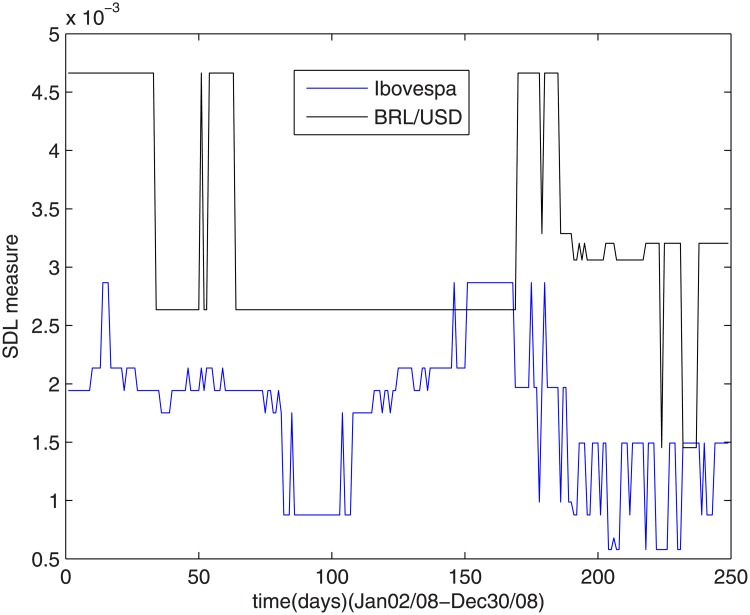
The SDL complexity measure during sub-prime crisis.

Paradoxically, the sub-prime crisis affected the Brazilian currency more than the Brazilian stock market, as shown in Fig 6.

During this period, the complexity measures related to Ibovespa index suffered small oscillations, maintaining low levels. Concerning the dollar/real ratio complexity, it became high with strong oscillations.

### 4.6 Dilma’s reelection

**Fig 7:**
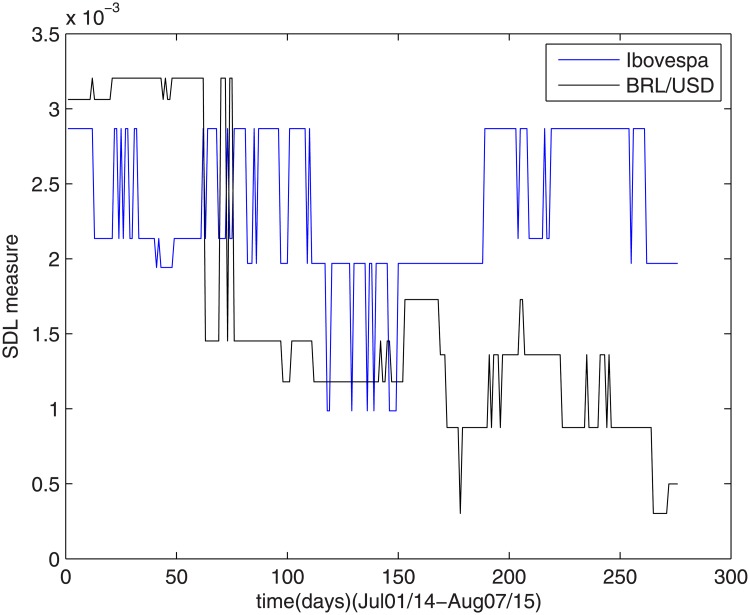
The SDL complexity measure during Dilma’s reelection.

During this period, both Ibovespa index and Dollar/Real ratio had their complexities strongly affected, as shown in Fig 7.

The measures presented high levels and oscillated atypically during this period.

## 5 Discussion

LMC and SDL complexity measures temporal series were defined for macro-economics variables. The definitions, applied to Brazilian economic data, have given some conjectures that can be divided into two groups: general methodological issues and particular diagnostic issues.

### 5.1 Methodological issues

The use of LMC and SDL complexity measures seem to show robust results, indicating that both are adequate to interpret macro-economic data. Additionally, either LMC or SDL might provide important qualitative hints about crisis events, being equivalent for data analysis.

Apparently, stock market indexes are more sensitive to the complexity calculations with results providing more evidence when crisis events occur. Consequently, to maintain a real time diagnostic of the complexity measure of these indexes can be an important diagnostic economic tool.

The complexity of currency indexes has given some diagnostics, but the robustness of the results is lower than the stock market complexity results with too many apparently spurious oscillations.

### 5.2 Brazilian crisis events

As Brazil is an important emerging economy, analyzing its data can give some hints regarding the social and political state of the country, as it was shown here.

For instance, the exchanging currency rule changes in 1999 produced an important instability of the stock market, measured by the high complexity of the Bovespa index, decreasing only in several months.

The twin-towers attack effects appeared in the high values of the Ibovespa index complexity, which remained high for several months after the event.

Lula’s campaign and election caused high complexity for the Ibovespa index, which remained for about 6 months after the elections, when the market understood that Lula was following a liberal policy.

The astonishing fact is that the sub-prime crisis has not strongly affected the Brazilian stock market, as shown by Ibovespa index complexity. The main motive is that the Brazilian market does not have complex derivative products. The effect appeared in the dollar-real ratio, probably because Brazilians suffer from a strong risk aversion.

Finally, when President Dilma Roussef initiated her reelection campaign, the Brazilian capital owners became apprehensive and started to declare that social actions were conducting the economy to bankruptcy. The newspapers and media owners agreed and a strong counter campaign spread throughout the country.

Regardless, Dilma Roussef won the elections, although the counter campaign continued, as it is evidenced by the complexity measures. Social scientists may tell the history in the next decades, with complexity measures helping in the analysis.

## 6 Conclusion

The LMC and SDL complexity measures provided some hints regarding Brazilian economy behavior. Although the work is exploratory, it can be useful to new analysis of economic data, attempting to explain how social and political events affect the economy.
